# One-Pot Green Synthesis of Amino Acid-Capped Gold Nanoparticles for Selective Sensing of Cyanide and Heavy Metals

**DOI:** 10.1021/acsomega.5c13624

**Published:** 2026-05-18

**Authors:** Beylem Girgin, Alper Baran Sözmen, Ahu Arslan-Yildiz

**Affiliations:** 52972 Izmir Institute of Technology (IZTECH), Department of Bioengineering, Engineering Building E, Izmir 35430, Turkey

## Abstract

In this study, 20 amino acids were utilized both as reducing and capping agents in a one-pot green synthesis of gold nanoparticles (GNPs) to be used in sensor applications for water, environment, and food monitoring. Tyrosine, tryptophan, valine, serine, phenylalanine, arginine, glutamic acid, and cysteine proved to be more suitable under the tested conditions, compared to the other amino acids, considering their colloidal stability. Amino acid-capped GNPs (AAGNPs) were then characterized in terms of absorbance spectrum, size, zeta potential, polydispersity index, geometry, and atomic content. After characterization, synthesized AAGNPs were utilized in sensory applications for Cyanide (CN^–^) and heavy metal (Al^3+^, Cu^2+^, and Fe^3+^) detection. The synthesized AAGNPs exhibited promising CN^–^ detection capability that is comparable to conventionally synthesized GNPs via the Turkevich method. Besides, ArgGNPs, GluGNPs, and CysGNPs exhibited distinct sensing behaviors, reflecting differences in surface chemistry and interaction mechanisms. Sensing platforms that utilized PheGNPs, TrpGNPs, and TyrGNPs showed detection limits in the range of 0.3–0.7 μM. Amino acid capping of GNPs imparted differential recognition capability toward various heavy metal ions, where detection limits were calculated for various AAGNPs as 0.27 mM for Cu^2+^ with CysGNPs, 0.25 mM for Al^3+^ with GluGNPs, and 0.44 mM for Fe^3+^ with SerGNPs. This phenomenon shows that synthesizing and capping GNPs with various amino acids not only alters the size and geometry, but also the capability of AAGNPs as parts of recognition elements of sensor systems. This study highlights the potential of amino acid-mediated green synthesis as an environmentally friendly and versatile approach for developing functional nanomaterials with tunable sensing capabilities for pollutant detection.

## Graphical abstract

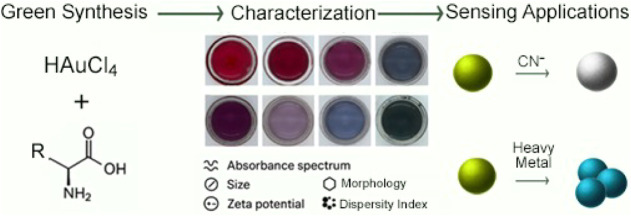

## Introduction

Gold nanoparticles (GNPs) have emerged as promising materials for biosensing applications due to their distinctive optical, electrical, and chemical properties.^1^ Various shapes and sizes of GNPs could be synthesized, and the synthesis can be achieved through complex strategies such as pH-dependent size control or simple strategies such as one-pot synthesis.^2–5^ GNPs have found diverse utility in the field of nanoscience, including material science, nanotechnology, and biomedical research, such as imaging, controlled delivery, diagnostics, and therapeutics.^6,7^ Conventional methods of fabricating nanoparticles have relied on the use of toxic chemicals as reducing and capping agents.^8–14^ For GNP synthesis, some commonly used approaches, such as citrate reduction, employ relatively benign reagents, while other established methods utilize strong reducing agents such as sodium borohydride and hydrazine, which are associated with safety concerns and the generation of undesirable byproducts.^15–18^ The Turkevich method of citrate reduction is among the most established approaches for relatively benign gold nanoparticle synthesis.^19^ In this context, green synthesis strategies have gained increasing attention as eco-friendly and sustainable alternatives.^6^ These approaches utilize biobased molecules, such as plant extracts, proteins, or amino acids, as both reducing and stabilizing agents.^20,21^ Amino acids offer structural diversity due to their functional groups (e.g., amine, carboxylate, thiol, and aromatic moieties), enabling modulation of reduction kinetics, surface binding strength, and interfacial chemistry. This allows tuning of nanoparticle size, morphology, and surface functionality compared to single-function reagents.^22–24^ In addition, amino acids can act as both reducing and capping agents, enabling simplified one-step synthesis approaches.^25^ Despite increasing interest in sustainable nanomaterial production, studies integrating amino-acid-mediated GNP synthesis with sensing applications remain limited. Existing work often focuses on synthesis optimization or structural characterization, rather than systematic evaluation of sensing performance in application-relevant contexts.^2,26–30^ This gap emphasizes the need for further studies to establish structure–function relationships and optimize GNP utility in sensitive and selective sensor platforms in terms of both synthesis and utilization.

Herein, the green synthesis of amino acid-capped gold nanoparticles (AAGNPs) was carried out using 20 essential amino acids. The synthesis was performed in a one-pot, single-step process, which eliminates the need for pre- and post-treatments, utilizing amino acids as both reducing and capping agents. The obtained GNPs were characterized using a UV–vis spectrophotometer, scanning electron microscopy (SEM), and dynamic light scattering (DLS). Out of the 20 amino acids, arginine, tyrosine, tryptophan, valine, serine, phenylalanine, glutamic acid, and cysteine were proposed to be utilized in sensor applications based on their stability, structure, and functionality. Then cyanide and metal ions were chosen as target analytes for proof-of-concept sensing applications since cyanide and metal ions such as Al^3+^, Cu^2+^, and Fe^3+^ are widely recognized as significant inorganic pollutants due to their environmental persistence and potential toxicity. In particular, Cu^2+^ and Fe^3+^ are often classified or considered heavy metal ions in environmental contexts because of their ecological impact and their ability to accumulate in natural systems. Cyanide is highly toxic even at lower concentrations because of its ability to inhibit cellular respiration.^31^ On the other hand, excessive copper and iron may lead to severe oxidative stress and organ damage when present above physiological limits, whereas excessive aluminum exposure has been associated with neurological and metabolic disorders.^32^ Therefore, rapid and accessible methods for monitoring these species are of considerable practical importance. Detection of said pollutants was carried out via measuring the change in the absorbance maximum value (λ_max_). Detection of CN^–^ was carried out leveraging the well-established principle that cyanide disrupts the GNP colloidal structure by forming soluble Au-CN complexes, which leads to a decrease in the λ_max_ value of GNPs.^33^ On the other hand, heavy metal detection was carried out through their interaction with AAGNPs, causing the formation of aggregates, which can be detected via a decrease in λ_max_. The performance of AAGNPs in sensor applications was compared with that of GNPs synthesized via the conventional Turkevich method. All the AAGNPs provided results similar to GNPs synthesized by the Turkevich method for CN^–^ detection, except for GluGNPs and CysGNPs. On the contrary, while conventionally synthesized GNPs were rendered incapable of heavy metal detection, CysGNPs, GluGNPs, and SerGNPs provided noteworthy results. This phenomenon shows that synthesizing and capping GNPs with various amino acids not only alters the size and morphology but also the behavior of GNPs. This diversity highlights the developed method and synhesized GNPs as promising alternatives to their conventional counterparts due to one-pot, eco-friendly, and single-step synthesis, as well as their potential for sensor applications.

## Results and Discussion

### Optimization of Synthesis Parameters

The synthesis protocol was optimized to obtain colloidally stable and geometrically uniform GNPs. The optimizations were carried out with the following parameters: HAuCl_4_ volume and concentration, amino acid volume and concentration, Tween-20 concentration, reaction time, and temperature (Table 1). Although 20 amino acids were initially screened as reducing and capping agents, only eight (Tyr, Trp, Val, Ser, Phe, Arg, Glu, and Cys) resulted in colloidally stable gold nanoparticle suspensions under the applied synthesis conditions. The remaining amino acids yielded less stable nanoparticles, likely due to a relatively lower reducing power or limited stabilizing capability. The selected amino acids represent the best-performing candidates across distinct chemical structures, such as acidic, basic, aromatic, or sulfur-containing residues, enabling systematic evaluation of structure and function relationships. The resultant AAGNP solution can be seen in Figure 1. The colors of the synthesized AAGNPs are as follows: TrpGNP is crimson red, TyrGNP is maroon, CysGNP is fuchsia, GluGNP is turquoise, PheGNP is purple, ValGNP is lilac, SerGNP is sky blue, and ArgGNP is dark green, whereas conventionally synthesized GNPs are characteristically wine-red, and related color codes can be seen in Table S1.

**Table 1:** Studied Ranges of Nanoparticle Synthesis Parameters

GNP	HAuCl_4_ concentration range (mM)	Amino acid concentration range (mM)	Tween-20 concentration range (% volume)	Temperature range (°C)	Reaction time range (min)
TrpGNP	0.31–0.63	0.1–6.0	0.01–0.1	50–100	5–40
TyrGNP	0.31–1.26	0.001–0.25	0.01–0.1	70–100	5–40
CysGNP	0.31–1.26	0.1–1.0	0.01–0.1	70–100	5–40
GluGNP	0.31–1.26	0.5–9.0	0.001–0.1	70–100	5–40
PheGNP	0.31–0.63	0.5–9.0	0.001–0.1	70–100	5–40
ValGNP	0.175–0.35	0.5–5.0	0.01–0.1	70–100	5–40
SerGNP	0.275–0.63	0.5–250	0.01–0.1	70–100	5–40
ArgGNP	0.31–2.52	0.5–62.50	0.001–0.1	70–100	5–40

**Figure 1:**
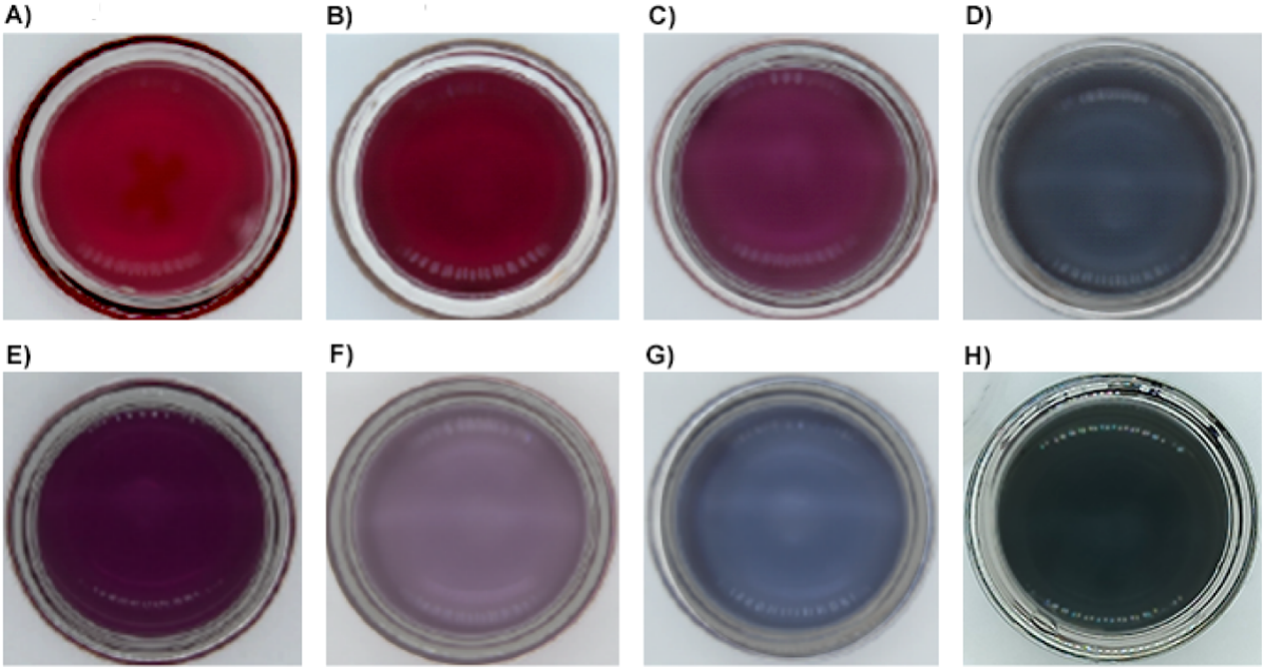
Final products of 8 GNPs synthesized: the colors of the GNPs are all different from each other. A) TrpGNP, B) TyrGNP, C) CysGNP, D) GluGNP, E) PheGNP, F) ValGNP, G) SerGNP, H) ArgGNP.

A total of 20 amino acids were utilized for synthesis, and 8 gave promising results in terms of ease of synthesis and colloidal stability afterward. These amino acids are Tyr, Trp, Val, Ser, Phe, Arg, Glu, and Cys, and their optimized synthesis parameters can be seen in Table 2. Once the amino acid concentrations were optimized, aggregation and accumulation of GNPs on used equipment were encountered for CysGNPs, TrpGNPs, and TyrGNPs. This issue was eliminated through temperature and reaction time optimizations, and where temperature adjustments were not sufficient, surfactant addition was considered, and out of the various surfactants, Tween-20 addition provided results with the least aggregation and accumulation. Hence, each AAGNP synthesis procedure required a separate optimization for the Tween-20 concentration.

**Table 2:** Optimized Synthesis Parameters of Each AAGNP

**GNP**	**HAuCl**_**4**_ **concentration (mM)**	**Amino acid concentration (mM)**	Tween-20 concentration (% volume)^a^	**Temperature (°C)**	**Reaction time (min)**
TrpGNP	0.63	2.00	0.02	50	10
TyrGNP	0.63	0.25	0.02	70	15
CysGNP	0.63	0.15	0.01	70	13
GluGNP	0.44	1.50	0.01	100	27
PheGNP	0.63	0.50	0.001	100	21
ValGNP	0.18	2.50	0.01	100	33
SerGNP	0.35	250.00	0.01	100	14
ArgGNP	1.26	15.60	0.01	100	30
ConGNP^b^	0.05	38.8	-	100	15

a Tween-20 was added postsynthesis in GluGNP.

b Conventionally synthesized GNPs via the Turkevich method.

All syntheses were performed under near-physiological pH conditions, where amino acid protonation states favor balanced reduction and stabilization. Experiments conducted outside this pH range consistently resulted in rapid aggregation, highlighting the critical role of pH in amino acid charge state, surface binding, and nanoparticle growth dynamics. These observations show that amino acid identity and solution chemistry jointly determine the nucleation–growth balance in green-synthesized gold nanoparticles.

### Characterization of AAGNPs in Terms of Size, Geometry, Zeta Potential, and Uniformity

Characterization of AAGNPs was initially performed using UV–Vis spectroscopy. The intensity and position of the localized surface plasmon resonance (LSPR) peak (λ_max_) were evaluated to obtain qualitative information about the optical behavior, dispersion state, and approximate size regime of the synthesized AAGNPs (Figure 2), since the optical properties of spherical gold nanoparticles are strongly influenced by their diameter.^34^ UV–Vis spectroscopy was used as the primary tool to guide the optimization of the nanoparticle synthesis, as it provides rapid and sensitive information on LSPR characteristics. During the optimization process, parameters such as the position of the plasmon band (λ_max_), intensity, and bandwidth were monitored as indicators of colloidal stability, aggregation state, and relative size distribution. In particular, plasmon band broadening is widely recognized as an indicator of increased polydispersity or nanoparticle aggregation in colloidal systems. Because the main objective of this study was to obtain stable plasmonic platforms suitable for sensing applications, the optimization focused on achieving reproducible optical properties rather than targeting a specific nanoparticle shape or size.

**Figure 2:**
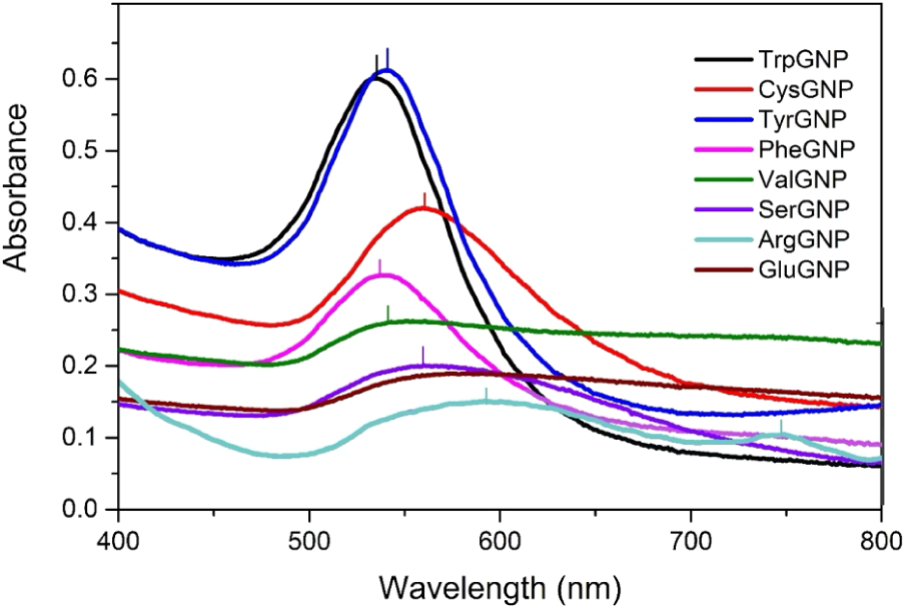
UV–vis absorbance graphs of 8 AAGNPs with corresponding λ_max_ locations of each peak are given.

For spherical gold nanoparticles at comparable mass concentrations, smaller particles typically exhibit LSPR maxima near 520 nm, whereas larger nanoparticles generally produce red-shifted and broader bands due to increased light scattering.^34,35^ This enhanced scattering arises from both larger optical cross-sections and a larger scattering to total extinction ratio. In the present study, all GNP samples exhibited λ_max_ values in the range of 535–560 nm, suggesting a size regime broadly consistent and might be around 40–100 nm.^36^ AAGNPs synthesized using amino acids with aromatic side chains produced relatively strong and narrow plasmon bands centered around 535–540 nm, which are often associated with well-dispersed colloidal systems with relatively narrow size distributions.^37^ On the other hand, SerGNPs displayed a broad, low-intensity peak, and a similar spectral behavior was observed for ValGNPs. Such features may suggest broader size distributions, partial aggregation, or the presence of anisotropic particle geometries.^38^ The absorbance spectrum of CysGNP exhibited a broader band with a λ_max_ at 541 nm compared with the systems containing aromatic amino acids, suggesting a comparable general size regime but potentially stronger surface interactions or increased polydispersity.^39^ Lastly, ArgGNPs exhibited two plasmonic peaks at wavelengths around 560 and 740 nm, which are generally attributed to urchin-shaped GNPs.^40^

It should be emphasized that plasmonic band positions should be interpreted as the indicators of the plasmonic properties, not as the sole indicators of size, since plasmonic response is also strongly influenced by particle geometry, anisotropy, and local dielectric environment.^41,42^ In particular, the presence of nonspherical or anisotropic structures, as observed by SEM for Tyr-, Glu-, and Val-capped GNPs, can induce significant red shifts even at smaller spherical diameters. The observed variations in plasmonic properties are therefore consistent with differences in particle size distribution and the possible formation of anisotropic nanostructures, in agreement with theoretical predictions based on Mie theory and discrete dipole approximation (DDA) models.^43,44^

The observed differences in optical response, particle geometry, and colloidal stability among AAGNPs can be attributed to differences in reduction behavior and surface binding interactions during nanoparticle formation.^25^ Amino acids influence nucleation and growth through their functional groups, which affect reduction kinetics and surface affinity. Aromatic amino acids, such as tryptophan, tyrosine, and phenylalanine, contain electron-rich π systems that may facilitate Au reduction. In particular, the indole group of tryptophan and the phenolic group of tyrosine have been reported to facilitate electron transfer processes, which can promote nucleation and stable colloid formation.^19^ Sulfur-containing cysteine exhibits strong affinity toward gold surfaces through Au–S interactions, which can lead to rapid surface passivation and nanoparticle growth.^45^ In contrast, acidic amino acids, such as glutamic acid, primarily contribute to electrostatic stabilization via carboxylate groups, while basic amino acids, such as arginine, show comparatively weaker reducing capability and may therefore produce less stable colloidal systems.^46^ Furthermore, the behavior of amino acids in such systems is strongly influenced by their protonation state, which depends on the reaction pH and can significantly affect both reduction pathways and surface binding interactions. These observations are consistent with reported mechanisms in the literature, where amino acid-dependent reduction and binding modes govern nucleation and growth.^19,39^ Consequently, nanoparticle properties reflect the combined effects of the reducing/capping agent, reaction conditions, reduction kinetics, and ligand binding affinity.

Further characterization of the synthesized AAGNPs was carried out using DLS to evaluate hydrodynamic diameter, zeta potential, and polydispersity index (PDI) values (Table 3). All samples exhibited PDI values in the range of 0.18–0.35, indicating relatively narrow to moderately broad size distributions depending on the amino acid used.^47^ In particular, TrpGNPs, TyrGNPs, ArgGNPs, and PheGNPs showed comparatively narrower distributions (PDI ∼ 0.18–0.24), whereas SerGNPs exhibited a higher PDI (∼0.35), suggesting increased size heterogeneity.^38^

**Table 3:** DLS Measurements of the Synthesized GNPs, Including Hydrodynamic Diameter, in Comparison with Dry-State Particle Sizes Obtained from SEM Image Analysis

**AAGNP**	**Poly dispersity index (PDI)**	**Potential (mV)**	Hydrodynamic diameter^a^ (nm)	**Dry-Phase diameter^b^ (nm)**
ArgGNP	0.235	25.43	37.2	25.8
TrpGNP	0.233	8.76	26.7	12.2
TyrGNP	0.188	–16.63	67.3	34.7
CysGNP	0.266	–5.93	78.8	62.0
GluGNP	0.273	–6.97	28.8	29.5
PheGNP	0.222	–24.15	31.7	40.7
ValGNP	0.232	–9.86	44.3	24.7
SerGNP	0.347	–19.04	36.7	16.6
GNP^c^	0.205	–58.44	22.4	18.4

a Values represent hydrodynamic diameters measured in aqueous suspension by DLS. The reported sizes correspond to the effective spherical diameter, which includes contributions from the solvation layers and surface-bound molecules. Therefore, these values may differ from the particle sizes observed in SEM analysis.

b Dry-phase particle size is measured via image analysis, for this purpose, at least 6 representative images and 60 randomly selected nanoparticles were utilized for mean diameter calculation for each AAGNP (*n* = 60).

c Synthesized via conventional Turkevich method, as control group.

DLS measurements revealed that the measured hydrodynamic diameters ranged from approximately 22 to 79 nm. Among the samples, CysGNPs (∼78.8 nm) and TyrGNPs (∼67.3 nm) displayed relatively larger hydrodynamic sizes, while ArgGNPs (∼37.2 nm), TrpGNPs (∼26.7 nm), and PheGNPs (∼31.7 nm) exhibited smaller particle sizes within the tens of nanometers range. ValGNPs (∼44.3 nm) and SerGNPs (∼36.7 nm) showed intermediate values, indicating moderate dispersion in solution. These results suggest that variations in hydrodynamic size are primarily influenced by differences in colloidal stability and the extent of particle association in suspension, rather than solely by primary particle size. It should be noted that DLS determines particle size based on translational diffusion and assumes an equivalent spherical particle model. Therefore, for anisotropic or irregularly shaped nanoparticles, the reported hydrodynamic diameter represents an effective size rather than the exact geometric dimensions of individual particles. This may lead to deviations when compared with SEM observations, particularly for samples exhibiting nonspherical morphologies or aggregation phenomena.^38,48^

In addition, ArgGNPs, CysGNPs, and PheGNPs exhibited relatively high absolute zeta potential values (|ζ| > 20 mV), indicating enhanced electrostatic stability, whereas TyrGNPs and SerGNPs showed moderately high values (|ζ| > 15 mV). ArgGNPs and TrpGNPs displayed positive zeta potentials, which may be attributed to the presence of amino acid functional groups on the nanoparticle surface. In the case of ArgGNPs, the positive surface charge is likely associated with the guanidinium functional group of arginine, which promotes strong electrostatic interactions and stabilization.^49–51^

Later, morphological characterization was performed using SEM analysis, where the acquired images revealed nanoparticle morphology and size features that were generally consistent with the DLS results (Figure 3 and Table 3, respectively). TrpGNPs (Figure 3B) exhibited relatively small and uniformly distributed particles with predominantly spherical morphology. Similarly, ArgGNPs (Figure 3A) and ValGNPs (Figure 3G) also appeared mainly spherical, with particle sizes in the tens of nanometers range, in agreement with their hydrodynamic diameters. In contrast, TyrGNPs (Figure 3C) and CysGNPs (Figure 3D) displayed relatively larger particle sizes along with a mixture of morphologies, including spherical, irregular, and anisotropic structures. GluGNPs (Figure 3E) also exhibited heterogeneous morphology, with both small particles and larger clustered structures visible within the same field of view. Such morphological diversity is known to influence plasmonic behavior and may contribute to broader UV–vis absorption features. In addition, partial aggregation observed in SEM images is attributed to drying effects, which may contribute to slight discrepancies between SEM-derived and DLS-measured sizes. On the other hand, PheGNPs (Figure 3F) showed a higher degree of aggregation in SEM images, forming clustered structures. This is attributed primarily to dry-state aggregation rather than representing the intrinsic particle size in solution, and therefore leads to relatively larger dry-phase diameter measurements compared to DLS.^52,53^

**Figure 3:**
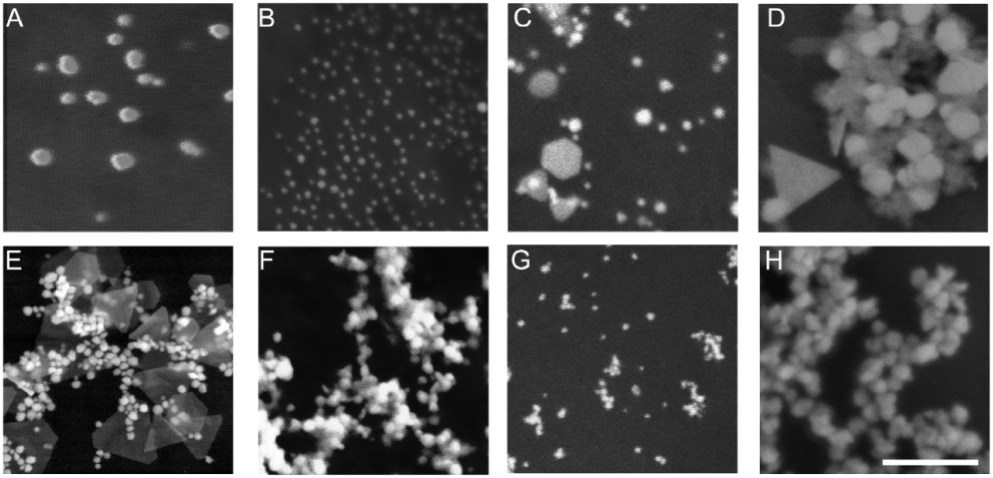
Representative SEM images of the 8 AAGNPs: A) ArgGNPs, B) TrpGNP, C) TyrGNP, D) CysGNP, E) GluGNPs, F) PheGNP, G) ValGNP, H) SerGNP. Scale bar: 250 nm. (Particle size ranges were determined from measurements of multiple particles across at least three independent SEM images and are summarized in Figure S1.)

Nanoparticle morphology was evaluated by using SEM, while size distribution and colloidal behavior in solution were assessed by DLS. Since the sensing mechanism in this study relies on solution-phase interactions and aggregation-induced optical responses, the hydrodynamic size and dispersion stability were considered more relevant than high-resolution structural imaging. Therefore, the combined use of SEM and DLS provides complementary information for confirming nanoparticle formation, morphology, and size distributions. Differences between hydrodynamic diameters obtained by DLS and particle sizes observed by SEM are expected because of the fundamental differences between these techniques. DLS measures the effective hydrodynamic diameter of particles in suspension, which includes contributions from solvation layers and dynamic aggregation, whereas SEM reflects the morphology and apparent size of dried particles, where aggregation may occur during the sample preparation.

To ensure accurate size interpretation, particle diameters were quantified from multiple SEM images, and the resulting size distributions are presented based on statistical analysis (Figure S1). The SEM-derived size ranges are generally consistent with the DLS measurements, with most samples exhibiting particle sizes within the tens of nanometer range. In some cases, larger structures observed in SEM images (e.g., GluGNPs, PheGNPs, and SerGNPs) are attributed to localized aggregation during drying and insufficient surface stabilization, while DLS measurements indicate relatively smaller hydrodynamic sizes in solution. Further stability profiles of the synthesized AAGNPs are shown in Figure S2. Overall, the revised dataset demonstrates good agreement between SEM and DLS results, supporting the formation of nanoscale particles with varying degrees of dispersion and aggregation depending on the amino acid used.

These observations indicate that optimal GNP synthesis and sensing performance require a balance between sufficient reduction potential to drive nucleation and appropriate surface binding to stabilize the growing nanoparticles. Amino acids with moderate redox capacity and functional groups that can weakly interact with gold are particularly well-suited to one-pot green synthesis protocols. Their resulting AAGNPs provide exposed and reactive surfaces, favorable for sensor applications based on surface-sensitive mechanisms. This structure–function relationship between amino acid chemistry and nanoparticle behavior highlights the potential for rational design of GNPs tailored for specific sensing applications by selecting capping agents based on their electrochemical properties and molecular structures. Herein, the sensing potential of AAGNPs was evaluated by CN^–^ and heavy metal detection, utilizing them both as recognition and transduction elements of a sensor system.

### Cyanide Detection Assay

Currently, the gold standard for cyanide detection is ion-selective electrode (ISE) analysis or spectrophotometric methods based on the Pyridine–Barbituric Acid (PBA) assay.^54^ These methods are reliable and accurate, but they require specialized laboratory equipment, skilled operators, and the usage of toxic organic solvents. This makes them less practical for rapid and on-site testing. In recent years, new colorimetric and fluorometric approaches for cyanide (CN^–^) detection have been developed, which are able to work without complex instruments and provide more environmentally friendly sensor platforms. Among these approaches, the utilization of GNPs for CN^–^ detection is a well-established procedure, hence, it was reenacted using AAGNPs synthesized in this study. CN^–^ ions can complex with gold atoms on GNP surfaces, forming soluble [Au­(CN)_2_]^−^ complexes and thereby disrupting the surface plasmon resonance, causing a decrease in λ_max_ intensity.^55,56^ Among all tested AAGNPs, TrpGNPs, TyrGNPs, and PheGNPs showed concentration-dependent decreases in λ_max_ intensity similar to conventional GNPs obtained by the Turkevich method. This behavior indicates that capping via these amino acids did not inhibit CN^–^ access to gold atoms, allowing efficient etching of the GNP surface, which shows strong potential for utilization in colorimetric CN^–^ sensors. Unexpectedly, CysGNPs showed an increase in absorbance upon CN^–^ exposure. This anomaly may result from the chemical interaction between CN^–^ and surface-bound cysteine. CN^–^ is known to cleave disulfide and thiol groups, yielding thiocyanate (SCN^–^) and regenerating free thiols.^57^ The formation of thiocyanate may stabilize the GNP surface against dissolution, or alter the dielectric environment, thereby increasing absorbance. On the other hand, GluGNPs exhibited visible color change upon CN^–^ exposure, yet no quantifiable decrease in λ_max_ value due to the absence of a defined UV–vis peak. This is consistent with the low optical signal-to-noise ratio arising from their polydispersity and broad NIR absorption.

As seen in Figure 4 most of the AAGNPs showed similar behavior to conventional GNPs, when CN^–^ was added, the only exceptions were CysGNP and GluGNP. Statistical evaluation of CN^–^ detection is given in Table S3. Also, Table 4 contains the LoD comparison of AAGNPs to conventional GNPs and correlation values between them. The lowest LoD values were acquired when PheGNPs were utilized, at 0.36 μM, followed by TrpGNPs and TyrGNPs with LoDs of 0.69 and 0.65 μM, respectively. Moreover, current gold standards utilized for CN^–^ detection provide an LoD in the range of 0.005–5 μM, which is comparable with the results acquired by AAGNPs^54^ and the permissible maximum value in water is determined to be 1.9 μM.^58^ The variation in synthesis efficiency, colloidal stability, and CN^–^ detection performance observed among different AAGNPs can be attributed to the distinct physicochemical properties of each amino acid.^25^ Overall, the responses, LoD values, and linearity of AAGNP sensing platforms demonstrated that PheGNPs, TrpGNPs, and TyrGNPs provide the best combination of sensitivity, stability, and optical response among green-synthesized candidates, for CN^–^ detection.

**Figure 4:**
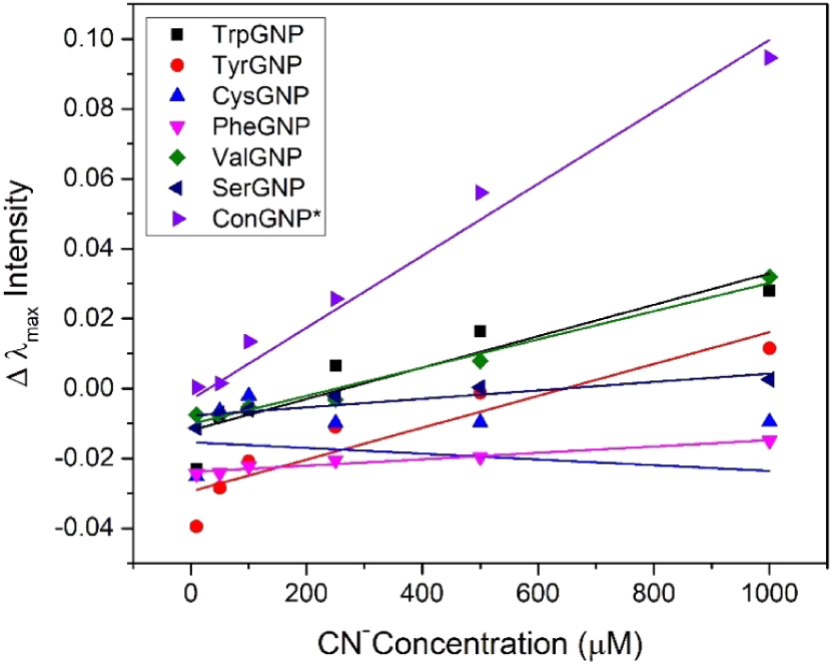
Calibration curves of various AAGNPs in comparison with conventionally synthesized GNPs.

**Table 4:** LoD Values of AAGNPs That Were Used for CN^–^ Detection and Their Correlation to Conventional GNPs

**AAGNP**	**LoD AAGNP (μM)**	**Correlation to conventional GNPs** (Pearson’s r)
TrpGNP	0.69	0.972
TyrGNP	0.65	0.979
PheGNP	0.36	0.980
ValGNP	2.35	0.955
SerGNP	0.85	0.951
ConGNP^a^	0.31	1

a Conventionally synthesized GNPs.

### Heavy Metal Detection Assay

In literature, the main detection techniques that are currently utilized for heavy metal detection include atomic absorption spectroscopy (AAS),^59^ furnace spectroscopy^60^ and inductively coupled plasma mass spectrometry (ICP-MS).^61^ These conventional techniques are highly sensitive; however, they lack portability, cost-effectiveness, and rapidness.^62^ Herein, metal detection was carried out employing developed AAGNPs following the same procedure in CN^–^ detection assay. Capping of GNPs with various amino acids allowed the functional groups to act both as recognition elements for metal ions. Upon the addition of specific heavy metal ions (Cu^2+^, Fe^3+^, and Al^3+^), the interaction between the metal ions and the surface-bound amino acids induces interparticle aggregation, leading to a characteristic red-shift in the absorbance peaks from ∼520 nm to ∼650–720 nm.^63,64^ The λ_max_ intensity decrease was used as an indicator of aggregation, which correlates linearly with metal ion concentration.

In some nanoparticle-ion combinations, minor visible color differences were observed after analyte addition. However, these changes were not uniform across all systems and did not provide a consistent qualitative indicator of response. Figure 5 shows calibration curves acquired by utilization of synthesized AAGNPs for heavy metal ion detection. Statistical evaluation of heavy metal detection is also given in Table S4. Among the tested AAGNPs, CysGNPs exhibited the highest sensitivity and selectivity toward Cu^2+^ ions. This response can be attributed to the thiol group in cysteine, which forms strong coordination bonds with Cu^2+^ promoting rapid aggregation.^65^ A significant decrease in λ_max_ intensity was observed within minutes of Cu^2+^ exposure, even at concentrations as low as 0.1 mM. CysGNPs also responded to Fe^3+^ ions with intensity comparable to Cu^2+,^ via thiol and carboxyl coordination, and showed minimal interaction with Al^3+^.^62^ On the other hand, GluGNPs demonstrated strong affinity for Fe^3+^ ions, which may be facilitated by its carboxyl groups, they act as oxygen donors to form stable chelates with Fe^3+^.^66^ Addition of Fe^3+^ resulted in a distinct decrease in λ_max_ intensity. Addition of Al^3+^ ions also induced aggregation in GluGNPs, although the overall response was weaker than that of Fe^3+^. These findings align with the known properties of heavy metal ions; as Fe^3+^ exhibits a high affinity for multiple carboxyl groups, and Al^3+^, being a strong Lewis acid, is more inclined to interact with oxygen donors.^67^ Furthermore, SerGNPs exhibited moderate decrease in λ_max_ intensity upon exposure to Fe^3+^ and Al^3+^, whereas Cu^2+^ induced negligible spectral changes, likely due to its lower affinity for oxygen donors, as noted in the literature.^68,69^ Hence, SerGNPs provide more selective detection for trivalent ions with lower sensitivity compared to cysteine or glutamic acid.^70,71^ Also, ArgGNPs showed moderate sensitivity toward Fe^3+^ and Al^3+^ ions, with aggregation likely mediated by the carboxyl and guanidinium functional groups.^72^ In contrast, PheGNPs showed minimal spectral changes upon exposure to any of the tested metal ions. The lack of strong polar functional groups on the phenylalanine side chain likely limits its ability to effectively coordinate with metal ions.^73^ On the other hand, conventionally synthesized GNPs showed no visible change in absorbance bands in the studied concentration range of metal ions. Overall, the observed selectivity patterns are consistent with established coordination chemistry principles in the literature.

**Figure 5:**
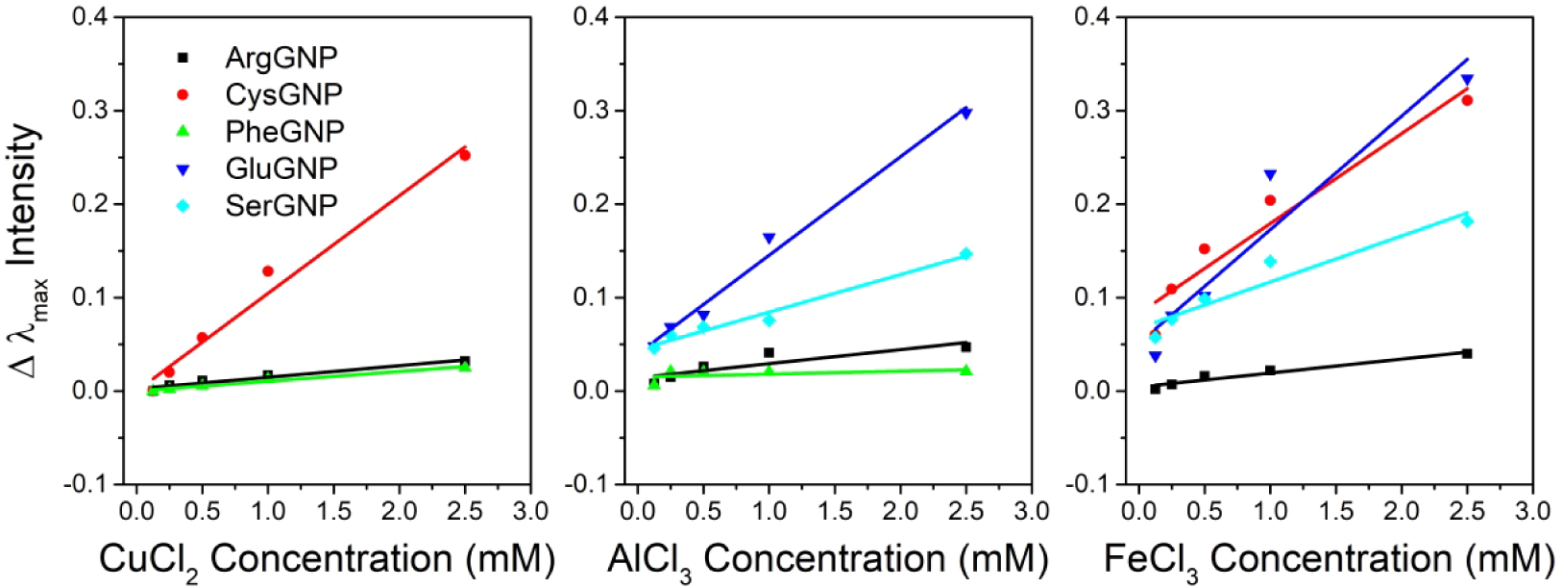
Calibration curves prepared utilizing the studied AAGNPs for detection of Cu^2+^, Al^3+^, and Fe^3+^.

SEM images acquired before and after heavy metal sensing are shown in Figure S3. They demonstrate that amino acid-functionalized gold nanoparticles exhibit ligand-dependent and size-dependent morphological transformations upon exposure to Cu^2+^, Al^3+^, and Fe^3+^ ions. CysGNPs showed the highest aggregation due to strong thiol–metal coordination, while GluGNPs formed interconnected networks, particularly in the presence of Al^3+^ through multivalent carboxylate bridging. SerGNPs displayed comparatively moderate structural changes, reflecting weaker hydroxyl–metal interactions. The SEM observations confirm that metal ion detection is primarily governed by surface coordination, interparticle cross-linking, and charge neutralization effects. These findings highlight the critical role of surface ligand chemistry and nanoparticle size in tuning selectivity and sensitivity for metal ion sensing applications.

The LoD values obtained for heavy metal ions fall within a concentration range that is higher than those reported for gold nanoparticle sensors specifically optimized for trace-level detection.^62,63^ Nevertheless, the maximum permissible limits of various heavy metals range between 0.05 and 2.3 mM.^62^ When these values are compared with the LoD values in Table 5, it is shown that AAGNPs utilized in heavy metal detection provided relevant LoDs in terms of real-life applications such as water, environmental, and food monitoring. More importantly, the present study emphasizes the possibility of a differential sensing behavior in a multiplexed sensing framework employing multiple AAGNPs with partially overlapping affinities, rather than maximum sensitivity toward a single analyte. These results demonstrate that simple amino acid capping of GNPs can impart differential recognition capabilities toward various heavy-metal ions, providing a low-cost, label-free platform for rapid spectrophotometric detection. The utilization of various amino acids in green-synthesized nanoparticle capping holds the potential for development into portable or multiplexed sensing platforms for environmental or biomedical monitoring of metal ions.

**Table 5:** LoD Values of Various AAGNPs When Utilized for Heavy Metal Detection (mM)

**AAGNP**	**CuCl**_**2**_	**AlCl**_**3**_	**FeCl**_**3**_
ArgGNP	0.66	1.34	1.42
CysGNP	0.27	N/a^a^	0.48
PheGNP	0.71	2.03	N/a^a^
GluGNP	N/a^a^	0.25	0.51
SerGNP	N/a^a^	0.85	0.44
ConGNP^b^	N/a^a^	N/a^a^	N/a^a^

a Not applicable.

b Conventionally synthesized GNPs.

The sensing experiments presented in this study are intended as a proof-of-concept demonstrating that amino acid-capped gold nanoparticles exhibit analyte-dependent optical responses arising from differences in surface chemistry and functional group composition. While CysGNPs, GluGNPs, and SerGNPs showed preferential responses toward Cu^2+^, Al^3+^, and Fe^3+^, respectively, comprehensive interference studies involving mixed-ion systems and common background cations (e.g., Na^+^, K^+^, Ca^2+^, Mg^2+^, Zn^2+^) were not exhaustively explored in this work. Rather than relying on strict one-to-one selectivity, the broader aim of this platform is the development of a multiplexed optical sensing array, utilizing multiple AAGNPs with partially overlapping affinities to generate characteristic response patterns, where current results show promising sensor array potential.

## Conclusion

In this study, a one-pot, green synthesis approach for fabricating amino acid-capped gold nanoparticles (AAGNPs) using eight different amino acids (Arg, Cys, Glu, Phe, Trp, Tyr, Ser, Val) as both reducing and capping agents was established. The synthesized AAGNPs were then utilized for pollutant detection, such as cyanide and heavy metals (Cu^2+^, Fe^3+^, and Al^3+^) as a cost-effective, rapid, and simple alternative to current methods. The synthesis conditions were optimized individually for each amino acid to maximize nanoparticle stability and yield, with Tween-20 employed as a surfactant to prevent aggregation. Comprehensive physicochemical characterization revealed significant variations in size, geometry, optical properties, and colloidal stability among the synthesized AAGNPs. PheGNPs displayed characteristics closest to those of conventionally synthesized GNPs, including a sharp plasmonic absorption band, a PDI of 0.27, and negative zeta potential (ζ < −20 mV). Also, SerGNPs appeared predominantly spherical, with particle sizes in the tens of nanometers range (∼30–40 nm) and a PDI value similar to PheGNPs. On the other hand ArgGNPs exhibited a positive zeta potential (ζ > 25 mV), and relatively small particle sizes around 30–40 nm, a PDI value of 0.251, and two distinct absorbance bands at 570 and 740 nm. Another notable AAGNP is GluGNPs, which exhibited a mixture of spheres, triangles, and irregularly shaped particles. It also showed a low PDI value of 0.25, suggesting that the observed morphological diversity is primarily influenced by synthesis conditions, although partial aggregation effects cannot be excluded. After characterization, AAGNPs-based sensor platforms were utilized for CN^–^ detection, and the results confirmed that, they exhibited comparable performance to conventional GNPs, particularly PheGNPs, TrpGNPs, and TyrGNPs. They showed concentration-dependent responses via λ_max_ intensity decrease, with the lowest LoD being 0.36 μM. Subsequently, the sensor platforms were used for heavy metal detection, with each AAGNP showing various affinities and differential recognition capabilities. Especially, CysGNPs, GluGNPs, and SerGNPs displayed distinct affinities for Cu^2+^, Fe^3+^, and Al^3+^ ions, governed by coordination chemistry, with LoD values of 0.27, 0.25, and 0.44 mM, respectively. This varied behavior of AAGNPs highlights the complexity of surface chemistry in functional nanomaterials and emphasizes the need for careful ligand selection in sensor design. Moreover, both sensor platforms showed dynamic detection ranges relevant to the maximum permissible limits of studied pollutants and in contrary to current gold standards, offered rapidness, portability, and cost-effectiveness. Further, the affinity variation of AAGNPs for different pollutants shows that the developed sensor platform also holds potential for multiplexed sensing. Overall, this work demonstrates the feasibility and versatility of amino acid-mediated green synthesis of GNPs for sensor development, offering an eco-friendly and scalable alternative to current labor-intensive methods that require expertise and laboratory infrastructure.

## Materials and Methods

### Materials

Gold­(III) chloride trihydrate (HAuCl_4_·3H_2_O) (Cat No: 520918, Sigma-Aldrich, USA), trisodium citrate (C_6_H_5_Na_3_O_7_) (Cat No: 1.11037, Sigma-Aldrich, USA), Tween-20 (BioShop, Canada), tryptophan, tyrosine, cysteine, phenylalanine, valine, glutamic acid, arginine, and serine (Chem Impex Int’l. Inc., USA), iron­(III) chloride anhydrous (FeCl_3_) (Cat No: 451649, Sigma-Aldrich, USA), aluminum chloride anhydrous (AlCl_3_) (Cat No: 563919, Sigma-Aldrich, USA), copper­(II) dihydrate (CuCl_2_·2H_2_O) (Cat No: 307483, Sigma-Aldrich, USA), and potassium cyanide (KCN) (Alfa Aesar, UK) were utilized in the experiments. All reagents were of analytical grade and used as purchased. Ultrapure water was used throughout all experiments.

### Gold Nanoparticle Synthesis

GNP synthesis was performed using a one-pot, green synthesis approach. Reaction conditions including concentrations of HAuCl_4_, amino acids, along with Tween-20 (as a stabilizer), also temperature and reaction time, were individually optimized for each amino acid as detailed below (Table 1). The parameter ranges were established through preliminary screening experiments using a one-factor-at-a-time approach, supported by literature-reported conditions for gold nanoparticle synthesis.^37,74,75^ This strategy was adopted to efficiently identify stable synthesis windows across multiple amino acid systems prior to detailed application studies. Although the onset of nanoparticle formation was initially indicated by a visible color change, reaction completion was determined objectively by UV–vis spectroscopy. Reactions were terminated once the absorbance at the λ_max_ reached a stable plateau, indicating the completion of nanoparticle formation.

The synthesis involved boiling HAuCl_4_, followed by the addition of amino acid solutions. Briefly, each AAGNP synthesis was started with heating the HAuCl_4_ solution to a boil and then adding the dissolved amino acids. Each reaction involved mixing aqueous HAuCl_4_ with the designated amino acid and Tween-20, followed by heating on a magnetic stirrer until a significant color change occurred (Figure 1).

In the synthesis of nanoparticles, the particle size, morphology, and colloidal stability can be altered by surfactants added to the aqueous medium. In this work, Tween-20 was used for stabilization and to prevent aggregation following the formation of the nanoparticles in an aqueous medium. Tween-20 is a nonionic, environmentally benign stabilizing agent that has been reported to be used previously in green synthesis approaches of nanomaterials.^76–78^

The concentration ranges selected for amino acids were determined after considering their reported solubility limits. Fresh stock solutions were prepared prior to synthesis under conditions ensuring complete dissolution, and working concentrations were chosen to remain well within the solubility range after the optimization studies.

Spectrophotometric measurements were carried out after each synthesis, and maximum absorbance intensity and narrow absorbance peak width were defined as optimization criteria. The Turkevich method was utilized as a control, 0.05 mM HAuCl_4_ solution was brought to 100 °C, then 38.8 mM trisodium-citrate was added, and the reaction was ceased after color formation.^74^

### Characterization of AAGNPs

In this study, UV–vis spectroscopy was employed to monitor the optical response of AAGNPs. The characteristic LSPR band arises from the collective oscillation of conduction electrons in response to light, which is highly sensitive to particle size, shape, and surface environment. However, it is important to emphasize that UV–vis does not measure physical dimensions directly; rather, it provides an indirect optical fingerprint that reflects underlying structural properties. Hence, a combination of UV–vis measurements with hydrodynamic size assessments from DLS and direct morphological observations from SEM was carried out to interpret the effects of particle geometry and dispersion, consistent with previous studies that demonstrated the utility of plasmonic spectroscopy for nanoparticle characterization, in a similar manner.

UV–vis absorbance spectrum characterizations were used during the optimization process for monitoring GNP synthesis and structures and sizes of the synthesized GNPs *(Thermo Scientific Multiscan GO)*.

SEM characterizations were carried out on an aluminum surface. Scanning Electron Microscope (SEM) (*FEI Quanta 250 FEG*) was used to acquire information in terms of nanoparticle’s geometry, chemical composition, and particle size. Particle size was measured via image analyses utilizing the software ImageJ/Fiji (NIH), at least 6 representative images and 60 randomly selected nanoparticles were utilized for mean diameter calculation for each AAGNP (*n* = 60).

Particle size, dispersity, and zeta potential characterizations were performed via dynamic light scattering (DLS) measurements using zeta Potential Analysis Instrument *(Malvern Panalytical’s Zetasizer).*

### Cyanide Detection Assay

Cyanide detection capability of synthesized AAGNPs was evaluated by monitoring absorbance changes after exposure to CN^–^. Cyanide solutions (10, 50, 100, 250, 500, and 1000 μM) were mixed with equal volumes of each AAGNP suspension and incubated at room temperature for 1 h (*n* = 6). UV–vis spectra were recorded before and after cyanide addition, and the change in absorbance intensity at λ_max_ (Δλ_max_) was used for detection.^54^

### Heavy Metal Detection Assay

Detection of Al^3+^, Cu^2+^, and Fe^3+^ was carried out utilizing synthesized AAGNPs as described above for CN^–^. Then, results were evaluated by monitoring λ_max_ changes after exposure to heavy metals. AlCl_3_, CuCl_2_, and FeCl_3_ solutions (10, 50, 100, 250, 500, and 1000 μM) were mixed with an equal volume of each AAGNP suspension and incubated at room temperature for 1 h (*n* = 6). UV–vis spectra were recorded before and after heavy metal addition, and the change in intensity at λ_max_ (Δλ_max_) was used for detection.^62,63^

### Statistical Analysis

Calibration curves were prepared using the mean signal value and plotted against concentration, and linear regression analysis was performed to obtain the calibration equation and correlation coefficient. LoD values were calculated using the 3σ/slope method, where σ corresponds to the standard deviation, and the slope was obtained from the linear region of the corresponding calibration curve.

## Supplementary Material



## Notes

Additional characterization; calibration data and regression statistics (standard error, *R* ^2^, Prob > *F*); electron microscopy-derived size distribution analyses; short-term stability study; supplementary control experiments and related SEM images for heavy metal detection (PDF)
