# Supplementary material for: One-Pot Green Synthesis of Amino Acid-Capped Gold Nanoparticles for Selective Sensing of Cyanide and Heavy Metals
Source: ACS Omega. 2026 May 18;11(21):30876–86. doi: 10.1021/acsomega.5c13624 (PMC13234877; doi:10.1021/acsomega.5c13624)
Supplement: Supplementary file 1 [file ao5c13624_si_001.pdf]

One-Pot Green Synthesis of Amino Acid-Capped Gold Nanoparticles for Selective Sensing of Cyanide and Heavy Metals

Beylem Girgin, Alper Baran Sözmen, Ahu Arslan-Yildiz**

Izmir Institute of Technology (IZTECH), Department of Bioengineering, Engineering Building E, 35430, Izmir, Turkey

Table S1. RGB Color codes of synthesized AAGNPs.

AAGNP	R	G	B
TrpGNP	125	0	33
TyrGNP	85	12	32
CysGNP	101	33	75
GluGNP	57	67	80
PheGNP	66	12	52
ValGNP	107	89	107
SerGNP	75	85	109
ArgGNP	33	45	45

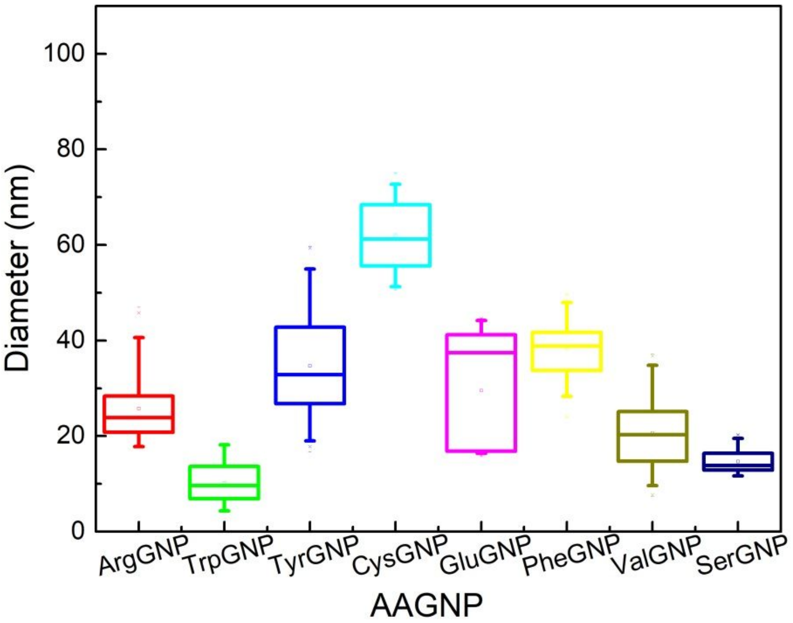

Figure S1. Particle size distributions of AAGNPs obtained from SEM image analysis. For each sample, particle diameters* were measured from multiple SEM images, and at least 60 particles per sample were analyzed (n=60) using image analysis software, ImageJ.

** The reported distributions represent the primary particle sizes and are used to support the qualitative observations from Figure 3 to compare with hydrodynamic diameters obtained from DLS measurements quantitatively.*

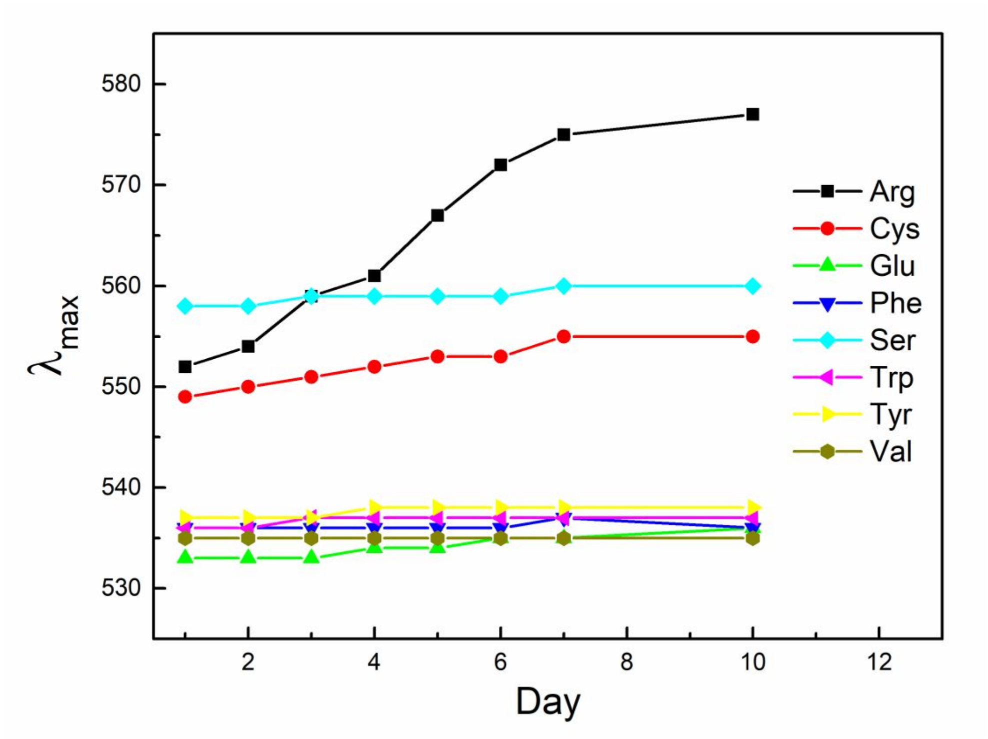

Figure S2. Stability profiles of synthesized AAGNPs, stored at +4°C.

Table S2. Atomic weight percentages of synthesized AAGNPs measured by EDX.

	Weight %				
AAGNP	Au	C	N	O	S
TrpGNP	20.35	54.63	10.80	14.18	
TyrGNP	37.85	46.56	1.63	13.96	
CysGNP	68.99	20.77	0.52	7.80	1.91
GluGNP	49.69	22.45	1.86	26.01	
PheGNP	52.81	22.48	4.76	19.95	
ValGNP	50.71	34.47	2.30	12.52	
SerGNP	19.84	37.62	23.14	19.40	
ArgGNP					

Table S3. Statistical evaluation of CN^- detection via AAGNPs

	**R Square**	**Std Error**	**Prob>F**
TrpGNP	0.83	0.007	0.011
TyrGNP	0.91	0.009	0.003
PheGNP	0.63	0.002	0.058
ValGNP	0.98	0.003	0.001
SerGNP	0.97	0.004	0.037

Table S4. Statistical evaluation of heavy metal detection via AAGNPs

	**$CuCl_2$**			**$AlCl_3$**			**$FeCl_3$**		
	**R Square**	**Std Error**	**Prob>F**	**R Square**	**Std Error**	**Prob>F**	**R Square**	**Std Error**	**Prob>F**
**ArgGNP**	0.93	0.002	0.004	0.70	0.005	0.051	0.93	0.002	0.005
**CysGNP**	0.97	0.009	0.001	-	-	-	0.92	0.015	0.007
**PheGNP**	0.97	0.001	0.001	-	-	-	-	-	-
**GluGNP**	-	-	-	0.98	0.007	0.001	0.90	0.020	0.010
**SerGNP**	-	-	-	0.96	0.003	0.002	0.89	0.050	0.011

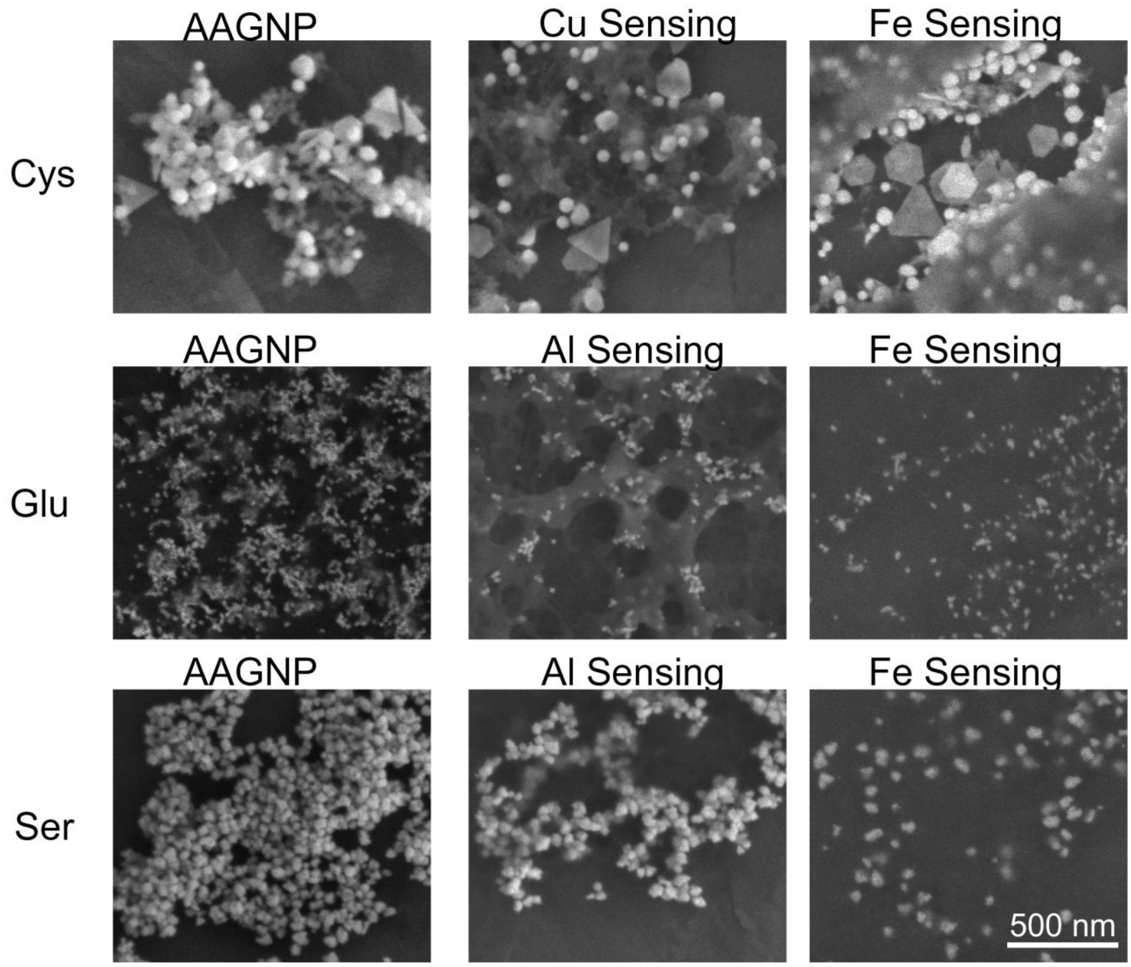

Figure S3.SEM images of AAGNPs taken before and after heavy metal sensing of various metals and AAGNP.
